# Supplementary material for: Echolocating toothed whales use ultra-fast echo-kinetic responses to track evasive prey
Source: eLife. 2021 Oct 26;10:e68825. doi: 10.7554/eLife.68825 (PMC8547948; doi:10.7554/eLife.68825)
Supplement: Transparent reporting form [file elife-68825-transrepform1.docx]

***eLife’s* transparent reporting form**

We encourage authors to provide detailed information *within their submission* to facilitate the interpretation and replication of experiments. Authors can upload supporting documentation to indicate the use of appropriate reporting guidelines for health-related research (see [EQUATOR Network](http://www.equator-network.org/%20)), life science research (see the [BioSharing Information Resource](https://biosharing.org/)), or the [ARRIVE guidelines](http://www.plosbiology.org/article/info:doi/10.1371/journal.pbio.1000412) for reporting work involving animal research. Where applicable, authors should refer to any relevant reporting standards documents in this form.

If you have any questions, please consult our Journal Policies and/or contact us: [editorial@elifesciences.org](mailto:editorial@elifesciences.org).

**Sample-size estimation**

- You should state whether an appropriate sample size was computed when the study was being designed
- You should state the statistical method of sample size computation and any required assumptions
- If no explicit power analysis was used, you should describe how you decided what sample (replicate) size (number) to use

Please outline where this information can be found within the submission (e.g., sections or figure legends), or explain why this information doesn’t apply to your submission:

Field experiments: The wild data used in this study were collected as part of previous projects and were re-analysed for the current study. Only data in which there were clear echo signatures during predator prey interactions could be used. The number of animals used in this study therefore represent a subsample of the available acoustic data. Clear echo signatures are vital to the identification of onset of prey movement during prey chases and this moment is the anchor from which we measure the onset of the predator response and so is essential for our measurements. 76 clear prey echo traces were identified across 6 harbour porpoise DTag deployments and 36 clear prey echo traces were identified across 7 beaked whale DTag deployments (See results section). The strong and stereotyped response signatures in these data following prey escape attempts indicate that sufficient data were available to characterise the behaviour of interest. However, the data are less suitable for the precise quantification of response latency and this led us to develop a controlled study.

Captive experiments: Harbour porpoises trained in the specific tasks needed to carry out these experiments, i.e., echolocating and approaching a specified target while carrying a suction-cup attached tag, are rare and therefore only two animals were used for the controlled study. In assessing the suitability of this study size, we reasoned that the reflex-like responses we expected to elicit should be displayed, in the right conditions, by all echolocating toothed whales. A similar number of animals have been used for other controlled studies that are testing a physiological process or deeply-seated behaviour that is expected to be shared by all individuals (e.g., Elmegaard et al., 2016 n=2, Wisniewska et al., 2015 n=3,).

Elmegaard, S.L., Johnson, M., Madsen, P.T. and McDonald, B.I., 2016. Cognitive control of heart rate in diving harbor porpoises. *Current Biology*, 26(22), pp.R1175-R1176.

Wisniewska, D.M., Ratcliffe, J.M., Beedholm, K., Christensen, C.B., Johnson, M., Koblitz, J.C., Wahlberg, M. and Madsen, P.T., 2015. Range-dependent flexibility in the acoustic field of view of echolocating porpoises (*Phocoena phocoena*). Elife, 4, p.e05651.

**Replicates**

- You should report how often each experiment was performed
- You should include a definition of biological versus technical replication
- The data obtained should be provided and sufficient information should be provided to indicate the number of independent biological and/or technical replicates
- If you encountered any outliers, you should describe how these were handled
- Criteria for exclusion/inclusion of data should be clearly stated
- High-throughput sequence data should be uploaded before submission, with a private link for reviewers provided (these are available from both GEO and ArrayExpress)

Please outline where this information can be found within the submission (e.g., sections or figure legends), or explain why this information doesn’t apply to your submission:

Sample size and experimental protocol are fully detailed in the “materials and methods” section of the manuscript.

In brief, for the two species of wild cetaceans, naturally-occurring predator-prey interactions were used as natural experiments which were recorded by biologging devices attached to the predator. Echogram visualisations of these data were examined to ensure that each interaction involved a unique prey which can therefore be expected to move independently and pose a fresh challenge to the tagged animal. Data were selected from all available prey capture attempts on the basis of clarity of echoic information and the presence of a clear escape reaction by the prey.

For trials using trained porpoises, up to 20 trials were performed with each animal per day, divided into a morning session and an afternoon session, resulting in a total of 150 trials over 4 days for the two animals. Responses to target movement were evaluated in 43 and 31 trials for Freja and Sif, respectively (as detailed in the results section of the manuscript). Responses during control trails (i.e., trials in which the target was not moved) were evaluated in 18 and 20 trials for Freja and Sif respectively.

Only trials in which a clear V-shaped echo trace was visible, due to the movement of the target relative to the porpoise, were included in the analysis. This was to maximise our ability to accurately align the time of target movement and the onset of the porpoise response. This meant that trials were excluded if the target did not attain a speed greater than the porpoises approach speed (approx., 1 m/s) (outlined in materials and methods section).

**Statistical reporting**

- Statistical analysis methods should be described and justified
- Raw data should be presented in figures whenever informative to do so (typically when N per group is less than 10)
- For each experiment, you should identify the statistical tests used, exact values of N, definitions of center, methods of multiple test correction, and dispersion and precision measures (e.g., mean, median, SD, SEM, confidence intervals; and, for the major substantive results, a measure of effect size (e.g., Pearson's r, Cohen's d)
- Report exact p-values wherever possible alongside the summary statistics and 95% confidence intervals. These should be reported for all key questions and not only when the p-value is less than 0.05.

Please outline where this information can be found within the submission (e.g., sections or figure legends), or explain why this information doesn’t apply to your submission:

Information on statistical analyses used are outlined in the materials and methods sections. In summary, to determine the probability of chance associations between prey/target movement and changes in biosonar sound production a bootstrapping method was applied to both wild and captive trials by randomly selecting, with replacement, 1000 pairs of trials and applying the elapsed time between key temporal markers (namely, the buzz start and prey/target movement) from one trial to assess the response in another trial (Outlined in materials and methods section). This controlled for temporal sequencing and chance associations that could lead to erroneous response detection. These faux trials differed from the true data in exactly the way expected. The results from this analysis for both wild and captive animals were so clear that little statistical interpretation was required. Raw results were accordingly presented in the form of simple box plots and no additional contrasts or tests were needed on these data.

To test the relationship between target acceleration and response magnitude during trials with trained animals in human care, a separate linear regression analysis was used for each animal (Outlined in Fig.7 legend). Raw data plots showing also the regression fit are provided for this analysis. Raw data are also shown in the main text and supplementary material to explain the processing steps.

(For large datasets, or papers with a very large number of statistical tests, you may upload a single table file with tests, Ns, etc., with reference to sections in the manuscript.)

**Group allocation**

- Indicate how samples were allocated into experimental groups (in the case of clinical studies, please specify allocation to treatment method); if randomization was used, please also state if restricted randomization was applied
- Indicate if masking was used during group allocation, data collection and/or data analysis

Please outline where this information can be found within the submission (e.g., sections or figure legends), or explain why this information doesn’t apply to your submission:

Our data allocation is outlined in the materials and methods section. For the wild data, species were treated separately but data from individuals within each species were grouped due to the small number of suitable trials. This is appropriate under the main hypothesis as we expect all animals to show a similar stereotyped response. The uniformity of the response in the grouped data supports this expectation. For captive animal trials, data from the two trained porpoises were treated separately as the goal here was to quantify response latency which may be expected to vary across individuals. For each animal, target movement was selected pseudo randomly for each trial between fast, slow, and no movement, with a maximum run of two equal conditions.

**Additional data files (“source data”)**

- We encourage you to upload relevant additional data files, such as numerical data that are represented as a graph in a figure, or as a summary table
- Where provided, these should be in the most useful format, and they can be uploaded as “Source data” files linked to a main figure or table
- Include model definition files including the full list of parameters used
- Include code used for data analysis (e.g., R, MatLab)
- Avoid stating that data files are “available upon request”

Please indicate the figures or tables for which source data files have been provided:

We have prepared a data archive and uploaded to this to Dryad. This is currently private but will be made public on acceptance of the manuscript. The complete source data for the paper comprise several TB, the great majority of which are not needed to reproduce our findings. We have therefore extracted a subset that is sufficient to re-create echograms for all trials and prey capture attempts of the type seen in Fig.2(A-B), Fig.3(A), Fig.4, Fig.5(A-B), and hence the results summary plots. Included for each trial is the acoustic data together with the timing of the prey/target movement and, for trials with trained animals, the speed of the target movement. Data comprises a single netcdf file for each analysed trial, and will cover the 112 captive trials (controls and treatment conditions) as well as each analysed prey capture attempt from the wild data. The netcdf format allows us to attach metadata directly to each trial in a human-readable form. Further we have provided Matlab/Octave scripts to produce echograms from these data. The supplementary material included in the manuscript provides sufficient information to interpret these echograms.
